# Risk of Gastrointestinal Bleeding with Rivaroxaban: A Comparative Study with Warfarin

**DOI:** 10.1155/2016/9589036

**Published:** 2016-01-06

**Authors:** Muhammed Sherid, Samian Sulaiman, Salih Samo, Husein Husein, Ruth Tupper, Charles Spurr, Humberto Sifuentes, Subbaramiah Sridhar

**Affiliations:** ^1^Section of Gastroenterology and Hepatology, Georgia Regents University, 1120 15th Street, AD 2226, Augusta, GA 30912, USA; ^2^Department of Internal Medicine, Froedtert Hospital and Medical College of Wisconsin, 9200 West Wisconsin Avenue, Milwaukee, WI 53226, USA; ^3^Department of Medicine, Northwestern Memorial Hospital, Northwestern University Feinberg School of Medicine, 251 East Huron Street, Suite 16-738, Chicago, IL 60611, USA; ^4^Department of Internal Medicine, Seton Hall University, School of Health and Medicine Sciences, Trinitas Regional Medical Center, 225 Williamson Street, Elizabeth, NJ 07202, USA; ^5^Department of Internal Medicine, Saint Francis Hospital, 355 Ridge Avenue, Evanston, IL 60202, USA

## Abstract

*Introduction*. The risk of gastrointestinal (GI) bleeding with rivaroxaban has not been studied extensively. The aim of our study was to assess this risk in comparison to warfarin. *Methods*. We examined the medical records for patients who were started on rivaroxaban or warfarin from April 2011 to April 2013. *Results*. We identified 300 patients (147 on rivaroxaban versus 153 on warfarin). GI bleeding occurred in 4.8% patients with rivaroxaban when compared to 9.8% patients in warfarin group (*p* = 0.094). GI bleeding occurred in 8% with therapeutic doses of rivaroxaban (>10 mg/d) compared to 9.8% with warfarin (*p* = 0.65). Multivariate analysis showed that patients who were on rivaroxaban for ≤40 days had a higher incidence of GI bleeding than those who were on it for >40 days (OR = 2.8, *p* = 0.023). Concomitant use of dual antiplatelet agents was associated with increased risk of GI bleeding in the rivaroxaban group (OR = 7.4, *p* = 0.0378). Prior GI bleeding was also a risk factor for GI bleeding in rivaroxaban group (OR = 15.5). *Conclusion*. The incidence of GI bleeding was similar between rivaroxaban and warfarin. The risk factors for GI bleeding with rivaroxaban were the first 40 days of taking the drug, concomitant dual antiplatelet agents, and prior GI bleeding.

## 1. Introduction

Over the last 60 years until very recently, vitamin K antagonists such as warfarin were the only oral anticoagulation agents available; however, due to their multiple drawbacks, the development of new oral direct factor inhibitors has emerged. Rivaroxaban is the first direct factor Xa inhibitor that has been approved by the US Food and Drug Administration (FDA) for the prevention of stroke and systemic embolism in patients with nonvalvular atrial fibrillation (AF), for the prevention and the treatment of venous thromboembolic events (VTE) [1–7]. It has also been tested for other indications [8–10]. The advantages of rivaroxaban over warfarin are numerous including a rapid onset of action, shorter half-life, no requirement for laboratory monitoring, minimal drug-drug and drug-food interactions, and minimal inter- and intraindividual variability with predictable anticoagulant effects and a fixed-dose regimen. Rivaroxaban dose varies depending on the indication and the risk of bleeding. It is administered as 10 mg daily (prophylactic dose) for VTE prevention after major joint surgeries, whereas a dose of 15 mg twice daily for 3 weeks followed by 20 mg daily is the regimen for the VTE treatment [2–7]. For nonvalvular AF, it is usually given as 20 mg daily; however a dose of 15 mg daily is administered in patients with a high risk of bleeding as in compromised renal function (GFR: 30–49 mL/min/1.73 m^2^) [1].

Rivaroxaban selectively inhibits the active site of factor Xa (both free and prothrombinase-bound forms) without a requirement for cofactors such as antithrombin III [11]. The oral bioavailability of rivaroxaban is approximately 80%, and the peak plasma concentration time is 1–4 hours [12–17]. Rivaroxaban has a dual mode of elimination; excreted by the liver and the kidney, thus, a marked impairment of the liver or renal function may affect its pharmacokinetics and pharmacodynamics [18–20]. Elimination half-life of rivaroxaban is 7–11 hours and extends up to 13 hours in the elderly [12–15, 17, 21, 22]. It has been proven that rivaroxaban has dose-dependent anticoagulation effects over a wide range of doses [12, 13]. Rivaroxaban is a substrate for P-glycoprotein and cytochrome P450 3A4; therefore, strong P-glycoprotein and cytochrome P450 3A4 inhibitors or inducers affect its pharmacokinetics and pharmacodynamics [23–25].

Despite these advantages of rivaroxaban over warfarin, the risk of bleeding, including gastrointestinal (GI) bleeding, is a concern with its use. Our hypothesis was that GI bleeding events occur more often in patients on rivaroxaban when compared to patients on warfarin. The primary aim of our study was to assess the risk of GI bleeding with rivaroxaban and compare to that of warfarin in a community hospitals setting and to identify the risk factors for developing GI bleeding in patients on rivaroxaban.

## 2. Methods

In this retrospective cohort study, we examined the medical records of all patients who were started on rivaroxaban from April 2011 to April 2013 and compared to a control group of consecutive patients who were started on warfarin during the same period in 1 : 1 fashion. The study was conducted in two hospitals (CGH Medical Center in Sterling, IL, and Saint Francis Hospital in Evanston, IL, USA) after obtaining the Institutional Review Board (IRB) approval from each institution. This study was conducted in collaboration with Georgia Regents University, Augusta, GA.

Demographic details, laboratory studies, information on the concomitant use of antiplatelet agents or nonsteroidal anti-inflammatory drugs (NSAIDs), duration of rivaroxaban and warfarin (≤ or >40 days), dose of rivaroxaban (10 mg/day (prophylactic dose), >10 mg/day (therapeutic dose)), indication for anticoagulation, GI bleeding events and the site of GI bleeding, major bleeding events and the site of bleeding, acute coronary syndrome (ACS), venous thromboembolic events (VTE), cerebrovascular events, and all-cause deaths while on an anticoagulation agent were collected. The primary aim was to assess the risk of GI bleeding in patients on rivaroxaban when compared to patients on warfarin during the study period and to identify any risk factors for developing GI bleeding. Gastrointestinal bleeding was defined as any bleeding in the GI tract that required hospitalization. The secondary aims were the risk of major bleeding events (defined as the bleeding events that required hospitalization and cessation of the anticoagulation agent), ACS that required angioplasty (with or without stenting) or heart surgery, VTE events (deep venous thrombosis or pulmonary embolism), cerebrovascular events (stroke or transient ischemic attack), and all-cause deaths while on an anticoagulation agent. Inclusion criteria were patients who were on rivaroxaban for ≥4 days during the study period and were compared to age and gender-matched patients who were on warfarin for ≥4 days. Exclusion criteria were an unknown duration of rivaroxaban or warfarin, lack of follow-up, age <18 years, pregnancy, mechanical valve replacement, and advanced kidney disease (glomerular filtration rate (GFR) < 15 mL/min/1.73 m^2^ or end-stage renal disease on dialysis).

### 2.1. Statistical Analysis

Patients' data were entered into Microsoft Excel spreadsheet in a coded format which was locked with a password. All analyses were performed with the use of SAS software (SAS Institute Inc., Cary, NC). A 2-sided *p* value of < 0.05 was considered statistically significant. Parametric two-sample *t*-tests were conducted to compare the means of the two groups involved, but the assumptions associated with *t*-tests (homogeneity of variances and normality of data) were not satisfied. Hence, the nonparametric Wilcoxon's rank sum test was conducted to compare the means of the two groups involved. A chi-square analysis, Fisher's exact test, and a Pearson correlation were conducted for the other variables. A logistic regression was carried out in multivariate analyses. Odds ratios (OR) were generated between group comparisons. A chi-square analysis was used for parameters in secondary aims.

## 3. Results

### 3.1. Patients (Table 1)

A total of 300 patients were identified, of whom 147 patients were on rivaroxaban (mean age 68.3 ± 14.5 years) and 153 patients were on warfarin (mean age 71.4 ± 13.1 years) (*p* = 0.0573) (Table 1). Eighty-five patients (57.82%) were ≥65 years in rivaroxaban group as compared to 104 patients in warfarin group (67.97%) (*p* = 0.0738). The majority of people in both groups were White (76.87% versus 86.93%) and female (52.38% versus 54.25%) in rivaroxaban and warfarin groups, respectively. Atrial fibrillation was the indication for anticoagulation in 46.94% in rivaroxaban group versus 60.78% in warfarin group (*p* < 0.0001). Prophylactic dose (10 mg/day) was used for VTE prophylaxis in 60 patients (40.82%) in rivaroxaban group, whereas therapeutic doses (>10 mg/day) were used in 87 patients (59.18%). The mean duration of drug use was significantly different between rivaroxaban and warfarin groups (92.19 ± 119.91 versus 252.95 ± 167.91, resp., *p* < 0.0001) with 55.1% of patients in rivaroxaban group being on the drug for ≤40 days when compared to only 10.46% of patients in warfarin group (*p* < 0.0001). Concomitant use of aspirin, thienopyridines (clopidogrel, ticlopidine, or prasugrel), dual antiplatelet agents (aspirin and thienopyridine), and NSAIDs was found in 38.10%, 8.16%, 5.44%, and 9.52%, respectively, in the rivaroxaban group when compared to 41.18%, 17.65%, 9.80%, and 4.58%, respectively, in the warfarin group (*p* values: 0.6372, 0.0163, 0.1558, and 0.1144, resp.). History of prior GI bleeding was present in 5 patients (3.40%) in the rivaroxaban group and 15 patients (9.80%) in the warfarin group (*p* = 0.0356). Laboratory studies (Hb < 12 g/dL, creatinine > 1.5 mg/dL, GFR ≤ 30 mL/min/1.73 m^2^, and ALT > 40 IU/dL) and BMI (<18.5, 18.5–24.9, ≥25 Kg/m^2^) were not significantly different between both groups (*p* > 0.05 for all parameters). We also separated the patients on therapeutic doses (>10 mg/day) of rivaroxaban from prophylactic dose group and compared them separately to the patients in warfarin group, to minimize the heterogeneity between both groups. The characteristics of patients on therapeutic doses are shown in Table 2.

Rivaroxaban was discontinued prematurely in 24 patients (16.33%) for various reasons (GI bleeding (7), major bleeding events (6), switching to warfarin for financial issue (6), high risk for bleeding (1), intolerance due to intractable vomiting (1), switch to dabigatran (1), switching to apixaban (1), and hospice (1)), whereas warfarin was discontinued prematurely in 28 patients (18.30%) for the following reasons (GI bleeding (15), major bleeding events (4), high risk for bleeding (4), switching to dabigatran (4), and switching to rivaroxaban (1)).

### 3.2. GI Bleeding Events (Table 3)

GI bleeding occurred in 7 patients (4.76%) in the rivaroxaban group when compared to 15 patients (9.80%) in the warfarin group (*p* = 0.094) (Table 3). All cases of GI bleeding in rivaroxaban group occurred in patients who were on therapeutic doses (>10 mg/day). As a result, 8.01% of patients on therapeutic doses of rivaroxaban developed GI bleeding which was not statistically different from warfarin group (*p* = 0.65). The mean age for GI bleeders in rivaroxaban group was 72.14 ± 15.40 years when compared to 75.80 ± 11.38 years in warfarin group (*p* = 0.4801). The mean duration of being on the drug was 29.00 ± 38.03 days in rivaroxaban group as compared to 163.87 ± 143.5 days in warfarin group (*p* = 0.0239). Concomitant use of antiplatelet agents or NSAIDs, laboratory parameters, BMI, and prior GI bleeding were not statistically different between GI bleeders in both groups (*p* > 0.05 in all parameters).

Multivariate analysis carried out using a logistic regression showed that patients who were on rivaroxaban for ≤40 days had a higher incidence of GI bleeding than those who were on the drug for >40 days (OR = 2.8, *p* = 0.023). Concomitant use of dual antiplatelet agents (aspirin and thienopyridine) was associated with an increased risk of GI bleeding in rivaroxaban group (OR = 7.4, *p* = 0.0378). A history of prior GI bleeding was a risk factor for GI bleeding in the rivaroxaban group (OR = 15.5). Age, gender, ethnicity, BMI, concomitant use of aspirin (alone), thienopyridines (alone), or NSAIDs, hemoglobin <12 g/dL, creatinine >1.5 mg/dL, GFR ≤ 30 mL/min/1.73 m^2^, and alanine aminotransferase >40 IU/L were not risk factors. The site of GI bleeding in rivaroxaban group was the lower GI tract in 57.14% and upper GI tract in 42.86% when compared to 33.33% and 40%, respectively, in the warfarin group with 26.67% without obvious site of GI bleeding (*p* > 0.05).  Table 4 shows the site of GI bleeding in both groups. There was no death related to GI bleeding in both groups.

### 3.3. Secondary Aims (Table 5)

Major bleeding events, defined as the bleeding events that required hospitalization and cessation of the anticoagulation agent, occurred in 6 patients (4.08%) in rivaroxaban group (hematuria (2), epistaxis (2), ICH (1), and postoperative knee {surgical site} bleeding that required reoperation (1)), when compared to 4 patients in warfarin group (retroperitoneal bleeding (2), hemarthrosis (1), and soft tissue bleeding due to valvular carcinoma (1)) (*p* = 0.5348). ACS that required angioplasty with or without stenting or heart surgery occurred in 3 cases (2.04%) in rivaroxaban group, compared to 8 cases (5.23%) in warfarin group (*p* = 0.2189). Symptomatic VTE events occurred in 2 cases in each group, and cerebrovascular events (stroke or TIA) occurred in one case of each group (*p* > 0.05 for both). There was no incidence of intracranial hemorrhage in warfarin group but there was one case in rivaroxaban group (*p* = 0.49). All-cause deaths while on the anticoagulation agent occurred in 1.36% in rivaroxaban group (Sepsis (2)) and in 5.23% in warfarin group (Sepsis (3), retroperitoneal bleeding (1), respiratory failure (1), congestive heart failure (1), lung cancer (1), and vulvar cancer (1)) (*p* = 0.1042). All the cases of secondary aim parameters occurred in patients on the therapeutic doses of rivaroxaban except 2 cases (one VTE and the case of postoperative knee bleeding) which occurred on the prophylactic dose.

## 4. Discussion

The most worrisome complication of anticoagulation agents is bleeding, including bleeding from the GI tract. There is scant information in the literature about GI bleeding in patients receiving the novel anticoagulation agent, rivaroxaban. This is the first retrospective comparative study to date that addresses the risk of GI bleeding with rivaroxaban as a primary aim of the study and identifies the risk factors for the development of GI bleeding when compared to warfarin.

In our study, GI bleeding occurred in none of 60 patients who were on the prophylactic dose (10 mg once daily) of rivaroxaban but all the events occurred on therapeutic doses (>10 mg/day). In the RECORD 1 trial, major GI bleeding occurred in 2 patients (0.09%) treated with prophylactic dose (10 mg/day) of rivaroxaban when compared to 1 (0.05%) in the enoxaparin group during the study period of 5 weeks for the thromboprophylaxis after hip arthroplasty [2]. One case of major GI bleeding (0.08%) occurred during the 5-week study period with 10 mg/day dose of rivaroxaban for thromboprophylaxis after hip arthroplasty in RECORD 2 [3]. This patient was on aspirin as well. There were no reports of major GI bleeding with the prophylactic dose of rivaroxaban during study period of 2 weeks for the thromboprophylaxis after total knee arthroplasty in RECORD 3 trial [4]. In RECORD 4, there was one case of fatal upper GI bleeding (0.07%) who was taking 2 types of NSAIDs and aspirin in rivaroxaban (10 mg daily) group during the 2-week study period when compared to none in enoxaparin 30 mg BID group [5]. In a pooled analysis of these four trials with a total of 12,729 patients receiving rivaroxaban (10 mg once daily) or enoxaparin, major GI bleeding occurred in 8 cases of 6183 patients (0.129%) on rivaroxaban when compared to one case in the enoxaparin arm (0.016%) during the 2 weeks, which comprised all cases of clinically overt extra-surgical-site bleeding events [26]. In MAGELLAN trial for extended thromboprophylaxis (35 ± 4 days) with 10 mg once daily dose of rivaroxaban in acutely ill hospitalized medical patients, there was a report of one case of fatal GI bleeding (0.025%) in the rivaroxaban group. However, GI bleeding events were not separated from other bleeding events in the published data [8].

As mentioned above, all cases of GI bleeding in our study occurred in patients on the therapeutic doses of rivaroxaban. It has been shown that major and nonmajor bleeding events, including GI bleeding, with rivaroxaban are dose-dependent as demonstrated in the phase II trials [27, 28]. In ODIXA-hip and ODIXA-knee phase II trials, the incidence of major bleeding events increased from 0.8–1% with a dose of 2.5 mg/BID to 5.4–7.5% with 30 mg/BID [27, 28]. There was also a significant dose trend for major bleeding in ODIXA-OD hip study with increased risk of major bleeding from 0.7% with a 10 mg once daily dose to 5.1% with 40 mg once daily dose during study period of 6–10 days [29]. Also, in ATLAS ACS-TIMI 46 dose-escalation phase II trials, the risk of clinically significant bleeding with rivaroxaban increased in a dose-dependent manner (hazard ratio increased from 2.21 with 5 mg dose to 5.06 with 20 mg dose) [9]. In EINSTEIN-DVT and EINSTEIN-PE studies, there was no separation of GI bleeding events from other major or nonmajor bleeding events in published data; however, in EINSTEIN-extension, there were 8 cases (1.34%) of GI bleeding events that described as clinically relevant nonmajor bleeding, but again there was no separation of major GI bleeding from other major bleeding events [6, 7].

Major GI bleeding events were more common in rivaroxaban group when compared to warfarin group in ROCKET AF study (3.15% versus 2.16%, resp., *p* < 0.0001) [1]. This risk was higher in those with moderate renal impairment (GFR of 30–49 mL/min/1.73 m^2^) when compared to those with preserved renal function, despite the dose reduction of rivaroxaban to 15 mg once daily instead of 20 mg once daily (4.1% versus 2.6%, resp., *p* = 0.02) [30]. In J-ROCKET AF Japanese trial which compared a Japan-specific reduced dose of rivaroxaban (15 mg once daily instead of 20 mg for those with preserved renal function and 10 mg once daily for those with GFR between 30 and 49 mL/min/1.73 m^2^ instead of 15 mg once daily) to warfarin, major GI bleeding events occurred in 8 cases (1.25%) in the rivaroxaban group and 15 cases in the warfarin group (2.35%) [31]. Majority of the bleeding occurred in the upper GI tract (6 cases versus 12 cases in rivaroxaban and warfarin, resp.) [31]. Of those 8 patients in the rivaroxaban group, 5 bleeding events occurred in patients with a preserved renal function when compared to 3 cases with GFR of 30–49 mL/min/1.73 m^2^ [32].

The number of GI bleeding events observed in our study was higher than the other studies, which may be attributed, in part, to the difference in the definitions of GI bleeding that was used in our study. We compared the incidence of all GI bleeding that required hospitalization and cessation of the active drug, as opposed to ROCKET AF trial which separated major GI bleeding (which was associated with dropping Hb by 2 g/dL or requiring transfusion of 2 units of blood) from nonmajor bleeding. By using our definition, it is possible that we included all GI bleeding events (major and nonmajor).

In our study, the first 40 days of rivaroxaban use was a risk factor for developing GI bleeding. The odds of GI bleeding for patients on rivaroxaban for ≤ 40 days were 2.8 times higher than those on the drug for >40 days. This is possibly because rivaroxaban may achieve the anticoagulant effect earlier than warfarin. The time in therapeutic range (TTR) for international normalized ratio (INR) for warfarin is low in the first months of starting the drug. For example, in EINSTEIN-DVT trial, the TTR was 54.1% in the first month when compared to 66.4% in the tenth month [6]. Rivaroxaban most likely unmasks the preexisting diseases (e.g., colon cancer) during the first 40 days of therapy faster than warfarin. Prior GI bleeding increased the risk of developing GI bleeding with rivaroxaban use by 15.5 times. This effect is again because of the rapid achievement of excellent anticoagulant effect and therefore rivaroxaban may unmask any preexisting GI condition.

The concomitant use of NSAIDs and antiplatelet agents with anticoagulants is important because of the potential increased risk of GI bleeding with their use; however, our study did not show any increased risk of GI bleeding with concomitant use of NSAIDs or aspirin (alone), or thienopyridines (alone). It has been shown that neither NSAIDs nor aspirin has a clinically relevant interaction with rivaroxaban [33, 34]. In RECORD trials, 72%, 9%, and <1% of patients used NSIADs (at least once), aspirin, and thienopyridines, respectively, during the study period which resulted in nonsignificant increases in any bleeding events [35]. Our study, however, showed that the concurrent use of dual antiplatelet agents (aspirin and thienopyridine) increased the risk of GI bleeding by 7.4 times. It has been shown that concomitant use of dual antiplatelet agents with anticoagulants, including novel oral anticoagulant agents such as rivaroxaban and dabigatran, increases the risk of major and nonmajor bleeding events including GI bleeding [36].

In our study, age, gender, ethnicity, BMI, hemoglobin of <12 g/dL, creatinine >1.5 mg/dL, GFR ≤ 30 mL/min/1.73 m^2^, and alanine aminotransferase >40 IU/L were not the risk factors for the development of GI bleeding in rivaroxaban group. However, the advanced age, especially the age over 75, was a risk factor for GI bleeding in patients on rivaroxaban in a recent study [37]. Similar to this study, Chang and his group using a medical and pharmacy administrative database demonstrated that the risk of GI bleeding was not different between rivaroxaban and warfarin which was similar to our study results [38]. The site of GI bleeding in the rivaroxaban group in our study was more often in the lower GI tract; however, it was not statistically different between the groups.

This study has several limitations. First, the size of the study was small when compared to other studies in this field. Second, since our study was retrospective, it carries its own bias such as missing information. Third, there was heterogeneity between both the groups such as age and previous GI bleeding which was minimized when only the therapeutic group was compared to the control group (compare Tables 1 and 2); however some other differences such as the indication for the drug continued which may have affected the results. Ideally, homogeneity between the studied group and control group is recommended; it is not always possible or feasible in a retrospective study without affecting the selection bias. The strengths in our study were that it reflects the real-world practice and also is the first study that addresses the risk of GI bleeding as a primary aim.

In conclusion, our study provides data that support the similarity of GI bleeding risks between rivaroxaban and warfarin. Rivaroxaban is more convenient than warfarin for both patients and health care providers. The risk factors for the development of GI bleeding in patients on rivaroxaban were the first 40 days of drug use, concomitant use of dual antiplatelet agents, and a history of previous GI bleeding. Our study is another step toward bid adieu to warfarin.

## Figures and Tables

**Table 1:** Demographic and clinical characteristics of patients in both groups.

Patients' characteristics	Rivaroxaban	Warfarin	*p* value
	(*N* = 147)	(*N* = 153)	
Mean age ± SD	68.25 ± 14.97	71.35 ± 13.09	0.0573

Age ≥ 65	85 (57.82%)	104 (67.97%)	0.0738
Age < 65	62 (42.18%)	49 (32.03)	

Gender			
Female	77 (52.38%)	83 (54.25%)	0.8170
Male	70 (47.62%)	70 (45.75%)	

Ethnic group			
White	**113 (76.87%)**	**133 (86.93%)**	0.0221^†^
AA	**14 (9.52%)**	**4 (2.61%)**	
Others	**20 (13.61)**	**16 (10.46%)**	

Indication for drug			
AF	**69 (46.94%)**	**93 (60.78%)**	**<**0.0001^†^
VTE treatment	**12 (8.16%)**	**56 (36.60%)**	
VTE prophylaxis	**60 (40.82%)**	**2 (1.31%)**	
Other	**6 (4.08%)**	**2 (1.31%)**	

Low dose	60 (40.82%)	NA	

High dose	87 (59.18%)	NA	

Mean duration being on drug (Days) ± SD	**92.19 ± 119.91**	**252.95 ± 167.91**	**<**0.0001^†^

Duration ≤40 days	**81 (55.10%)**	**16 (10.46%)**	**<**0.0001^†^

Concomitant with aspirin	56 (38.10%)	63 (41.18%)	0.6372

Concomitant with thienopyridine	12 (8.16%)	27 (17.65%)	0.0163

Dual antiplatelet agents	8 (5.44%)	15 (9.80%)	0.1558

Concomitant with NSAIDs	14 (9.52%)	7 (4.58%)	0.1144

Hb < 12	55 (39.29%)	54 (35.29%)	0.5454
Missing data	7	0	

Cr > 1.5	6 (4.23%)	15 (9.80%)	0.0714
Missing data	5	0	

GFR ≤ 30	2 (1.41%)	6 (3.92%)	0.2851
Missing data	5	0	

ALT > 40	**11 (9.91%)**	**28 (20%)**	0.0349^†^
Missing data	**36**	**13**	

BMI			
<18.5	5 (3.52%)	0 (0%)	0.0707
18.5–24.9	31 (21.83%)	35 (22.88%)	
>25	106 (74.65%)	118 (77.12%)	
Missing data	5	0	

Previous GI bleeding	**5 (3.40%)**	**15 (9.80%)**	0.0356^†^

AA: African American, AF: atrial fibrillation, ALT: alanine aminotransferase, BMI: body mass index, Cr: creatinine, GFR: glomerular filtration rate, GI: gastrointestinal, Hb: hemoglobin, NSIADs: nonsteroidal anti-inflammatory drugs, and VTE: venous thromboembolism events. ^†^Signifying statistical significant values.

**Table 2:** Demographic and clinical characteristics of patients of therapeutic dose group of rivaroxaban compared to warfarin.

Patients' characteristics	Therapeutic dose group of rivaroxaban	Warfarin	*p* value
	(*N* = 87)	(*N* = 153)	
Mean age ± SD	69.94 ± 15.24	71.35 ± 13.09	0.7088

Age ≥ 65	59 (67.82%)	104 (67.97%)	0.98
Age < 65	28 (32.18%)	49 (32.03)	

Gender			
Female	39 (44.83%)	83 (54.25%)	0.1605
Male	48 (55.17%)	70 (45.75%)	

Ethnic group			
White	66 (75.86%)	133 (86.93%)	0.285
AA	12 (13.79%)	4 (2.61%)	
Others	9 (10.35%)	16 (10.46%)	

Indication for drug			
AF	**67 (77.01%)**	**93 (60.78%)**	**<**0.0001^†^
VTE treatment	**12 (13.79%)**	**56 (36.60%)**	
VTE prophylaxis	**2 (2.30%)**	**2 (1.31%)**	
Other	**6 (6.90%)**	**2 (1.31%)**	

Mean duration being on drug (Days) ± SD	**125.30 ± 131.00**	**252.95 ± 167.91**	**<**0.0001^†^

Duration ≤40 days	**31 (35.63%)**	**16 (10.46%)**	**<**0.0001^†^

Concomitant with aspirin	**44 (50.58%)**	**63 (41.18%)**	0.0008^†^

Concomitant with thienopyridine	11 (12.64%)	27 (17.65%)	0.3074

Dual antiplatelet agents	8 (9.20%)	15 (9.80%)	0.8776

Concomitant with NSAIDs	8 (9.20%)	7 (4.58%)	0.2187

Hb < 12	27 (32.14%)	54 (35.29%)	0.0803
Missing data	3	0	

Cr > 1.5	5 (5.95%)	15 (9.80%)	0.1134
Missing data	3	0	

GFR ≤ 30	1 (1.20%)	6 (3.92%)	0.5645
Missing data	3	0	

ALT > 40	8 (10.26%)	28 (20%)	0.1081
Missing data	9	13	

BMI			
<18.5	3 (3.45%)	0 (0%)	0.4152
18.5–24.9	21 (24.14%)	35 (22.88%)	
>25	63 (72.41%)	118 (77.12%)	
Missing data	0	0	

Previous GI bleeding	4 (4.60%)	15 (9.80%)	0.1551

AA: African American, AF: atrial fibrillation, ALT: alanine aminotransferase, BMI: body mass index, Cr: creatinine, GFR: glomerular filtration rate, GI: gastrointestinal, Hb: hemoglobin, NSIADs: nonsteroidal anti-inflammatory drugs, and VTE: venous thromboembolism events. ^†^Signifying statistical significant values.

**Table 3:** Demographic and clinical characteristics of patients with GI bleeding in both groups.

Characteristics of patients with GI bleeding	Rivaroxaban	Warfarin	*p* value
	(*N* = 7)	(*N* = 15)	
GI bleeding events	7/147 (4.76%)	15 (9.80%)	0.094

GI bleeding events with high dose	7/87 (8.01%)	15 (9.80%)	0.65

Mean age ± SD	72.14 ± 15.40	75.80 ± 11.38	0.4801

Gender			
Female	2 (28.57%)	7 (46.67%)	0.4214
Male	5 (71.43%)	8 (53.33%)	

Ethnic group			
White	6 (85.71%)	13 (86.67%)	0.9517
AA	1 (14.29%)	1 (6.67%)	
Others	0	1 (6.67%)	

Indication for drug			
AF	5 (71.43%)	10 (66.67%)	0.8233
VTE treatment	1 (14.29%)	5 (33.33%)	
VTE prophylaxis	1 (14.29%)	0	
Other	0	0	

Prophylactic dose	0	NA	

Therapeutic doses	7	NA	

Mean duration being on drug (days) ± SD	**29.00 ± 38.03**	**163.87 ± 143.5**	0.0239^†^

Duration ≤40 days	5 (71.43%)	5 (33.33%)	0.3770

Concomitant with aspirin	4 (57.14%)	7 (46.67%)	0.6471

Concomitant with thienopyridine	3 (42.86%)	4 (26.67%)	0.4476

Dual antiplatelet agents	2 (28.57%)	3 (20%)	0.655

Concomitant with NSAIDs	1 (14.29%)	0	0.1341

Hb < 12	3 (42.86%)	7 (46.67%)	0.8673

Cr > 1.5	1 (14.29%)	2 (13.33%)	0.9517

GFR ≤ 30	0 (0%)	0 (0%)	NA

ALT > 40	1 (14.29%)	2 (13.33%)	0.9517

BMI			
<18.5	0	0 (0%)	0.899
18.5–24.9	3 (42.86%)	6 (40%)	
>25	4 (57.14%)	9 (60%)	

Previous GI bleeding	2 (28.57%)	1 (6.67%)	0.1632

Upper GI tract	3 (42.86%)	6 (40%)	0.899

Lower GI tract	4 (57.14%)	5 (33.33%)	0.29

Occult GI bleeding	0 (0%)	4 (26.67%)	0.1309

Death related to GI bleeding	0 (0%)	0 (0%)	NA

AA: African American, AF: atrial fibrillation, ALT: alanine aminotransferase, BMI: body mass index, Cr: creatinine, GFR: glomerular filtration rate, GI: gastrointestinal, Hb: hemoglobin, NSIADs: nonsteroidal anti-inflammatory drugs, and VTE: venous thromboembolism events. ^†^Signifying statistical significant values.

**Table 4:** Site of GI bleeding.

Site of GI bleeding	Rivaroxaban (*n*)	Warfarin (*n*)
Upper GI tract	(a) Distal esophageal ulcer with friable ulcer (1) (b) Gastric ulcer (1) (c) Esophageal varices (1)	(a) PUD (2) (b) AVM in stomach/duodenum (3) (c) Sphincterotomy site after ERCP (1) (d) Scopes were not performed (1)^‡^

Lower GI tract	(a) Rectal cancer (1) (b) Internal hemorrhoids (1) (c) Internal hemorrhoids with diverticulosis and multiple colonic polyps (1) (d) Sigmoid ulcers (1)	(a) Colon cancer (2) (b) Internal hemorrhoids (c) Large cecal polyp (1) (d) Scopes were not performed (1)^*∗*^

Occult GI bleeding	None	EGD/colonoscopy/push enteroscopy were negative (4)

AVM: arteriovenous malformation, EGD: esophagogastroduodenoscopy, ERCP: endoscopic retrograde cholangiopancreatography, and PUD: peptic ulcer disease. ^‡^Patient had hematemesis which indicated upper GI bleeding, but scopes were not performed because of patient's refusal. ^*∗*^Patient had bright red blood per rectum which indicated lower GI bleeding in the clinical scenario, but scopes were not performed because of patient's refusal.

**Table 5:** Secondary aims.

Events	Rivaroxaban	Warfarin	*p* value
	(*N* = 147)	(*N* = 153)	
Major bleeding other than GI bleeding	6 (4.08%)	4 (2.61%)	0.5348
ICH	1 (0.68%)	0 (0%)	0.4900
Stroke or TIA	1 (0.68%)	1 (0.65%)	1.0000
Symptomatic VTE	2 (1.36%)	2 (1.31%)	1.0000
ACS that required interventions	3 (2.04%)	8 (5.23%)	0.2189
All-cause death	2 (1.36%)	8 (5.23%)	0.1042

ACS: acute coronary syndrome, ICH: intracranial hemorrhage, TIA: transient ischemic attack, and VTE: venous thromboembolism events.
